# Trauma-Related Psychopathology in Iraqi Refugee Youth Resettled in the United States, and Comparison With an Ethnically Similar Refugee Sample: A Cross-Sectional Study

**DOI:** 10.3389/fpsyg.2021.574368

**Published:** 2021-03-22

**Authors:** Lana Ruvolo Grasser, Luay Haddad, Suzanne Manji, Shervin Assari, Cynthia Arfken, Arash Javanbakht

**Affiliations:** ^1^Stress, Trauma, and Anxiety Research Clinic (STARC Lab), Department of Psychiatry and Behavioral Neurosciences, Wayne State University, Detroit, MI, United States; ^2^Department of Family Medicine, Charles R. Drew University of Medicine and Science, Los Angeles, CA, United States; ^3^Department of Urban Public Health, Charles R. Drew University of Medicine and Science, Los Angeles, CA, United States

**Keywords:** mental health, psychiatry, psychological stress, refugees, developmental psychology, anxiety, PTSD, trauma

## Abstract

**Background:**

Conflict in Iraq has displaced millions of refugee youth. Warzone exposure and forced migration have unique acute and chronic impacts on youth, yet effects of exposure may not be universal across diverse refugee groups. Understanding how youth from various refugee groups are differentially affected by stress and trauma is critical to allocate resources and implement screening measures with the goal of providing early intervention.

**Method:**

To identify the effects of warzone exposure and forced migration, a convenience sample of 48 Iraqi refugee youth ages 6–17 was assessed within the first month of arrival to the United States. Youth provided self-reported severity of posttraumatic stress and anxiety symptoms; symptom severity was then compared with an existing sample of 135 Syrian refugee youth to explore whether refugee youth of different nationalities experience the same effects of warzone exposure and forced migration. These data are the baseline for a longitudinal developmental study of refugee health, which also includes parental data.

**Results:**

Severity of separation anxiety and negative alterations in cognition and mood were the greatest symptomatic concerns in Iraqi refugee youth. Thirty-eight percent of responding Iraqi youth showed possible indication of an anxiety disorder. Severity of posttraumatic stress symptoms was lower in Iraqi youth compared to Syrian youth. For both Iraqi and Syrian refugee youth, separation anxiety was the most significant concern, with more than 80% of both samples showing a possible indication of clinically significant separation anxiety.

**Conclusion:**

The present observational study indicated that Iraqi refugee youth experience a range of anxiety and posttraumatic stress symptoms following warzone exposure and forced migration; posttraumatic stress symptoms were less severe in Iraqi versus Syrian youth. Comparing refugee youth of different nationalities is of particular importance, as our results demonstrate that findings from one refugee population cannot easily be generalized to another. Clinical and research efforts should prioritize interventions to address separation anxiety in refugee youth, which was of concern in both samples.

## Introduction

The ongoing conflict in Iraq (2003–present) has resulted in the displacement of more than 3 million people (UNHCR, 2018). These individuals have been forced to flee their countries because of persecution, war, or violence – defining refugee status (UNHCR, 1984). Trauma exposure and subsequent psychopathology in child and adolescent refugees is an important area to explore, as childhood trauma exposure has significant acute impact, as well as long-term effects (Cohen et al., 1993). Additionally, understanding the potential differences between refugees originating from different countries is necessary given refugees’ experiences may vary based on nationality, ethnicity, religion, and reason for fleeing.

Thirty percent of Syrian refugee youth resettled in Germany screen positive for PTSD (Soykoek et al., 2017); in the United States, 52.9% screen positive for anxiety (Javanbakht et al., 2018). Seven percent of adult Iraqi refugees in the United States have been exposed to primary torture; 45.9% have experienced secondary torture (Willard et al., 2014). Trauma-related psychopathology is less clearly defined in Iraqi refugee youth, and the present study fills this gap by assessing severity of trauma-related psychopathology (posttraumatic stress and anxiety symptoms) in Iraqi refugee youth resettled in the United States. Observations from one refugee group, such as Syrian refugee youth, may not necessarily generalize to another group, such as Iraqi refugee youth. For instance, there are different levels of stress among Muslim and non-Muslim Iraqi adult refugees (Arfken et al., 2018), and female gender and religion of Islam (compared to Buddhism and Christianity) are predictors of high anxiety in a cohort of torture survivors arriving in the United States (Song et al., 2015; Abu Suhaiban et al., 2019). The present study compares effects of warzone exposure and forced migration between similar youth refugee cohorts.

We previously published methods for collecting self-report questionnaire data regarding symptom severity and possible indication of PTSD, anxiety, and depression in Syrian adult (Javanbakht et al., 2019) and child refugees (Javanbakht et al., 2018) and in Iraqi adult refugees (Arfken et al., 2018) in this context. The primary aims of this study were to (1) assess severity posttraumatic stress and anxiety symptoms, as well as possible indication of PTSD and anxiety disorders, in Iraqi refugee youth resettling in Southeastern Michigan, and (2) compare levels of distress in the present Iraqi refugee youth with a sample of Syrian refugee youth resettled in the same region at the same time period. This is specifically important as these refugees’ may seem similar in terms of the timing and type of trauma and postmigration circumstances. To achieve these aims, we administered self-report questionnaires measuring posttraumatic stress and anxiety symptoms to refugee youth within 1 month of their arrival in the United States at primary care clinics. Our goal was to provide an indication of the effects of stress and trauma on Iraqi refugee youth, which may require focused treatment and to explore whether the effects of stress and trauma vary between refugee youth of two different nationalities.

## Methods

The State of Michigan has resettled the second highest number of Iraqi refugees between 2002 and 2018, only behind California, with 1,119 Iraqi refugees resettling in 2016 alone (Warikoo, 2018). The present study population represents a convenience sample of Iraqi refugees presenting to a local primary care clinic run by the Arab American Chaldean Council (ACC) for mandatory physicals within their first month of arrival to the United States. Iraqi refugee families were informed by clinicians of the opportunity to participate in voluntary, paid research study. Those who were interested were then transferred to another room to meet the study team and provide consent for participation in research. Written assent was obtained from all subjects/patients between ages 13 and 17, with corresponding written parental consent. Verbal consent was witnessed and formally recorded for all subjects/patients ages 6–12, with corresponding written parental consent. Inclusion criterion was as follows: (1) male and female Iraqi refugee youth between the ages of 6 and 17. Exclusion criteria were as follows: (1) unaccompanied minors, (2) wardens of the court, and (3) past or current diagnosis of psychosis. The authors assert that all procedures contributing to this work comply with the ethical standard of the relevant national and institutional committees on human experimentation and with the Helsinki Declaration of 1975, as revised in 2008. All procedures involving human subjects/patients were approved by the Institutional Review Board at Wayne State University.

Newly arriving Iraqi refugee families were referred to the bilingual research team by primary care physicians following their mandatory medical screening. Families were informed of a paid volunteer research opportunity they could participate; they were also informed and ensured that their participation in the research study would not affect any participation or care at the clinic. Interested families were then introduced to the research team and were provided with consent forms explaining the study. Data consisted of self-report questionnaires administered in Arabic, available in verbal or written form for ease and convenience of participants. Questionnaires administered in Arabic had been translated from English to Arabic by a native speaker, and back-translated to English by a separate Arabic speaker to ensure consistency and accuracy in translations. Translations were certified and approved by external reviews. Questionnaires were in paper format and were filled out by either the participant him/herself or the clinical research assistant conducting the interview with the participant if this additional assistance was needed. These included a demographics questionnaire, the UCLA PTSD RI (Steinberg et al., 2013), and the SCARED (Birmaher et al., 1997) for children. The UCLA PTSD RI and SCARED provide an indication of the extent of possible posttraumatic stress and anxiety disorders, but are not structured clinical interviews. Study procedures were identical for the population of Syrian refugee youth that we have previously published on Javanbakht et al. (2018). Data collection for both populations occurred simultaneously.

Severity of posttraumatic stress symptoms and possible indication of PTSD were assessed using the UCLA PTSD Reaction Index for Children and Adolescents (UCLA PTSD RI) for DSM 5. The UCLA PTSD RI is a 31-item questionnaire to which participants indicate how much they have been bothered by particular symptoms within the last month, ranging from none of the time (0) to most of the time (4). The first 27 items on the questionnaire map onto the four symptom clusters, with the last four questions assessing the dissociative subtype. Possible indication requires one or more category B (intrusion) symptom present, one or more category C (avoidance) symptom present, two or more category D (negative alternations in cognitions and mood) symptoms present, two or more category E (arousal and reactivity) symptoms present, symptom duration greater than month, and symptoms causing clinically significant distress or impairment. A symptom is “present” if the symptom score is 3 or 4, with the exceptions of questions 4, 10, and 26, where a rating of 2 or more is indicative of symptom presence (Steinberg et al., 2004, 2013). The UCLA PTSD RI has been recommended for use with refugee populations, including those with non-Western backgrounds (Miller et al., 2019), and has been found to be reliable (Javanbakht et al., 2018). Cronbach’s alpha for the UCLA in this sample was.946, representing a high degree of internal consistency.

Severity of anxiety symptoms and possible indication of an anxiety disorder was assessed using the Screen for Child Anxiety Related Emotional Disorders (SCARED). The SCARED is a 41-item questionnaire to which participants indicate whether each item describes them (very true – 2 or often true – 1) or not (0). A total score of 25 or higher is indicative of a possible anxiety disorder; subscales are then used to determine possible presence of panic disorder/significant somatic concerns (score of 7 or higher), generalized anxiety disorder (GAD; score of 9 or higher), separation anxiety (score of 5 or higher), social anxiety (score of 8 or higher), and significant school avoidance (score of 3 or higher) (Birmaher et al., 1997, 1999). The SCARED has been recommended for use with refugee populations, including those with non-Western backgrounds (Miller et al., 2019), and has been shown to be valid and reliable in non-Western samples (Su et al., 2008; Essau et al., 2013; Javanbakht et al., 2018). Cronbach’s alpha for the SCARED in this sample was.925, representing a high degree of internal consistency.

All analyses were performed in R version 4.0.3. Data were analyzed by a researcher who was not involved in data collection and was blinded to subject identifiers to reduce any potential bias. Standard data screening was performed for all variables. The median response for a scale was used to fill missing questionnaire data when two or fewer responses were missing; otherwise, the questionnaire was considered as “missing” for the individual. Following scoring of questionnaires, missing data were then imputed for total and subscale scores for all scores, which had less than 50% missing data using eight iterations of multiple imputation. Outliers were corrected with winsorization. To compare sex differences in severity of symptoms for Iraqi refugee youth, a Wilcoxon rank sum test with continuity correction was applied as the non-parametric test equivalent of the independent samples *t* test. This method was selected due to the small sample of Iraqi refugee youth. To compare impact of refugee-related trauma in Syrian and Iraqi youth, a one-way ANOVA with type III sum of squares was used to accommodate the unbalanced design, as there was a greater number of Syrian than Iraqi youth. Possible indications of PTSD and anxiety disorders were considered dichotomous variables and not included in statistical models; instead, we compared symptom severities as continuous variables to reflect mental health on a continuum in accordance with Research Domain Criteria initiatives.

## Results

### Demographics

Demographic characteristics are presented in Table 1. Data were obtained from 48 Iraqi refugee youth (21 girls; 27 boys) between the ages of 6 and 17, mean age = 11. The majority of participants arrived with both parents, rated their health as “excellent”, and reported a lack of English speaking and writing fluency. Five youth reported a medical condition, including ulcer, autism spectrum disorder, and asthma. One of these youth reported being on medication. Half of the sample reported religion of Islam; half reported Christianity. There were no differences in symptom severity based on religious affiliation. The present dataset reflects single timepoint baseline data from a larger longitudinal study of Syrian and Iraqi refugee youth, which also includes parental data, expanded reporting with ongoing data collection, and will include an added comparison group of non-refugee Middle Eastern immigrant youth.

**TABLE 1:** Participant demographics [*n* = 183 (48 Iraqi; 135 Syrian)].

Measure	*n*, Iraqi	%, Iraqi	*n*, Syrian	%, Syrian
Sex				
Male	27	56.2	73	54.1
Female	21	43.8	59	43.7
**“How would you rate your overall health?”**				
Excellent	32	78.0	57	54.8
Very good	5	12.2	30	28.8
Good	4	9.8	17	16.4
Fair	0	0	0	0
Poor	0	0	0	0
**Medical condition**				
Yes	5	10.6	9	7.0
No	42	89.4	120	93.0
**Medication use**				
Yes	1	2.2	6	5.1
No	45	97.8	111	94.9
**Cigarette use**				
Yes	0	0	1	0.8
No	48	100	119	99.2
Hookah use				
Yes	1	2.1	1	0.8
No	47	97.9	119	99.2
**English speaking level**				
Not at all	20	64.5	33	38.3
Not well	7	22.6	47	54.7
Well	3	9.7	6	7.0
Very well	1	3.2	0	
**English writing level**				
Not at all	18	58.1	35	41.2
Not well	5	16.1	43	50.6
Well	8	25.8	7	8.2
Very well	0	0	0	0

*No participants reported cannabis, opioid, cocaine, or stimulant use. No participants reported exposure to chemical attacks.*

### Self-Reported Severity of PTSD and Anxiety in Iraqi Refugee Youth

Thirty-eight percent of Iraqi youth indicated a possible anxiety disorder based on the total SCARED score (see Table 2). For specific anxiety disorders, separation anxiety was the most highly prevalent condition with 87.5% of youth screening positive; all but three boys and three girls in the sample screened positive for separation anxiety (see Table 2). Four youth screened positive for PTSD based on the UCLA PTSD RI; 9.5% of the sample; three were boys. Posttraumatic stress symptoms were relatively low; cluster C symptoms (negative alterations in cognition and mood; “cognition and mood”) were the most elevated in this sample (see Table 3). Rates of possible PTSD and anxiety disorders, broken down by sex, are presented in Table 2. Symptom severity was not significantly correlated with age for any measure.

**TABLE 2:** Possible indications for PTSD, an anxiety disorder, and specific anxiety disorders broken down by nationality and sex.

Measure	Y/N, Iraqi	% +, Iraqi	Y/N, Syrian	% +, Syrian
**Possible PTSD**				
Male	3/21	12.5	5/45	10.0
Female	1/17	5.5	1/31	3.1
**Possible anxiety Dx**				
Male	4/7	36.4	33/40	45.2
Female	4/7	36.4	34/25	57.6
**Possible panic/somatic Dx**				
Male	3/8	37.5	9/64	8.2
Female	3/8	37.5	14/45	25.4
**Possible GAD**				
Male	2/9	18.2	12/61	16.4
Female	3/8	37.5	6/53	10.2
**Possible separation anxiety**				
Male	24/3	89.9	60/13	82.2
Female	18/3	85.7	47/12	79.7
**Possible social anxiety**				
Male	5/8	38.5	22/51	30.1
Female	2/9	18.2	16/43	27.1

*Anxiety and specific anxiety disorders were assessed using the self-report SCARED questionnaire. Possible indication of any anxiety disorder is defined by the SCARED as a total sum score of 25 or greater. Possible prevalence of panic disorder/somatic problems, GAD, separation anxiety, and social anxiety defined by the SCARED are subscale sum scores of 7, 9, 5, and 8, respectively. PTSD was assessed using the self-report UCLA PTSD RI. Possible prevalence of PTSD was based on published scoring guidelines for the UCLA, which are in accordance with DSM-5 definition of PTSD.*

**TABLE 3:** Descriptive statistics for severity of total anxiety symptoms and specific anxiety symptoms as a function of nationality (country of origin).

Symptom	Nationality	*M*	*M*	SD
			95% CI	
			[LL, UL]	
Total anxiety	Syrian	22.78	(21.49, 24.07)	7.58
	Iraqi	22.35	(16.85, 27.86)	12.41
Panic/somatic	Syrian	3.63	(3.14, 4.11)	2.84
	Iraqi	3.94	(2.33, 5.55)	3.64
GAD	Syrian	4.86	(4.35, 5.36)	2.97
	Iraqi	5.06	(2.94, 7.19)	4.79
Separation anx.	Syrian	8.19	(7.59, 8.78)	3.50
	Iraqi	7.63	(6.94, 8.31)	2.36
Social anx.	Syrian	5.48	(4.91, 6.05)	3.35
	Iraqi	5.58	(3.78, 7.39)	4.27

*Self-reported symptom severities were assessed using the SCARED. *M* and SD represent mean and standard deviation, respectively. LL and UL indicate the lower and upper limits of the 95% confidence interval for the mean, respectively. The confidence interval is a plausible range of population means that could have created a sample mean (Cumming, 2014).*

### Comparison of Iraqi and Syrian Refugee Youth

Next, we compared our sample of Iraqi youth to our sample of Syrian youth, which has been previously published (Javanbakht et al., 2018) and was composed of *n* = 135 (ages 5–17, *m*_*age*_ = 11, 59F). Demographic characteristics of the Syrian youth sample are presented alongside those of the Iraqi youth sample in Table 1. The sample characteristics were relatively similar, such that presence of medical conditions and use of medication as well as substances were minimal, and youth from both samples had similarly experienced warzone exposure and forced migration, arriving in the United States at the same time and resettling in the same region. In both samples, measured sociodemographics were not significantly related with severity of symptoms (see Tables 3, 4 for symptom severities descriptive statistics by nationality). One-way ANOVAs for unbalanced samples showed no significant differences in age or self-reported severity of anxiety, panic/somatic symptoms, GAD, separation anxiety, social anxiety, English speaking fluency, or English writing fluency (Table 5). Total posttraumatic stress symptoms were significantly more severe in Syrian youth than Iraqi youth; more specifically, re-experiencing, avoidance, and cognition/mood symptoms were higher for Syrian youth compared to Iraqi youth (Table 6). Additionally, self-reported perception of health was significantly worse in Syrian youth than in Iraqi youth, *F*(1,143) = 4.96, *p* = 0.028, *d* = 0.411. It should be noted that in the Syrian cohort, there were no significant differences in symptom severity by sex, as was also the case for the Iraqi cohort.

**TABLE 4:** Descriptive statistics for severity of total posttraumatic stress symptoms and specific symptoms clusters as a function of nationality (country of origin).

Symptom	Nationality	*M*	*M*	SD
			95% CI	
			[LL, UL]	
Total PTSS	Syrian	15.82	(14.01, 17.63)	10.65
	Iraqi	11.04	(8.10, 13.98)	10.14
Re-experiencing	Syrian	2.75	(2.30, 3.19)	2.62
	Iraqi	1.60	(0.92, 2.29)	2.37
Avoidance	Syrian	0.95	(0.69, 1.21)	1.52
	Iraqi	0.94	(0.51, 1.36)	1.46
Cognition/mood	Syrian	7.27	(6.29, 8.24)	5.72
	Iraqi	5.39	(3.79, 6.98)	5.50
Arousal	Syrian	3.28	(2.73, 3.82)	3.20
	Iraqi	3.00	(2.08, 3.92)	3.15
Dissociation	Syrian	1.01	(0.76, 1.27)	1.49
	Iraqi	0.79	(0.40, 1.18)	1.35

*Self-reported symptom severities were assessed using the UCLA PTSD RI. *M* and SD represent mean and standard deviation, respectively. LL and UL indicate the lower and upper limits of the 95% confidence interval for the mean, respectively. The confidence interval is a plausible range of population means that could have created a sample mean (Cumming, 2014).*

**TABLE 5:** One-way ANOVA for unbalanced samples comparing severity of anxiety symptoms in refugee youth of Iraqi and Syrian nationalities.

Symptom	*F*	*df*	*p*	Cohen’s *d*	*Post hoc* power
Total anxiety	0.05	1,155	0.827	0.050	0.175
Panic/somatic	0.22	1,155	0.643	–0.107	0.124
GAD	0.08	1,155	0.783	–0.064	0.155
Separation anx.	1.06	1,181	0.306	0.173	0.129
Social anx.	0.02	1,157	0.896	–0.029	0.097

*Self-reported symptom severities were based on the SCARED. *Post hoc* power analyses were performed in R using simulations based on the present sample sizes, means, and standard deviations for each measure. Results presented herein should be interpreted with caution, given the lack of statistical power for most comparisons due to the limited sample.*

**TABLE 6:** One-way ANOVA for unbalanced samples comparing severity of posttraumatic stress symptoms in refugee youth of Iraqi and Syrian nationalities.

Symptom	*F*	*df*	*P*	Cohen’s *d*	*Post hoc* power
Total PTSS	7.30	1,181	0.008	0.454	0.769
Re-experiencing	7.08	1,181	0.009	0.447	0.768
Avoidance	0.00	1,181	0.966	0.007	0.051
Cognition and Mood	3.90	1,181	0.049	0.332	0.499
Arousal/Reactivity	0.27	1,181	0.606	0.087	0.081
Dissociation	0.83	1,181	0.363	0.153	0.126

*Self-reported symptom severities were based on the UCLA PTSD RI. *Post hoc* power analyses were performed in R using simulations based on the present sample sizes, means, and standard deviations for each measure. Results presented herein should be interpreted with caution, given the lack of statistical power for most comparisons due to the limited sample.*

## Discussion

In this work, we examined the impact of civilian war trauma exposure and forced migration on Iraqi refugee youth resettling in Southeastern Michigan. Thirty-eight percent of Iraqi youth in our sample screened positive for an anxiety disorder, higher than that of the general United States population – 28.8% (Kessler et al., 2005). The youth in our sample fall at the average age of onset (11) for anxiety disorders (Kessler et al., 2005), with a mean age of 11, so in tandem with the trauma exposure and stress of forced migration, it is not surprising to see anxiety as a noticeable psychiatric concern in this cohort. While we present the incidence of possible anxiety reported in our limited sample of Iraqi refugee youth alongside the general United States population rate, direct comparison should not be made between these two studies, given the epidemiological study involved in a very large, randomly selected, representative sample.

Separation anxiety was the greatest concern, with 87.5% of the Iraqi refugee youth sample presenting with a possible indication. This finding is particularly noteworthy as daily life functioning – including social and educational success – relies on the ability for youth to attend school, interact with peers, participate in group activities, and form social networks. Behaviors such as not separating from immediate family members and staying within the home or other safe settings are adaptive in dangerous situations like a warzone. Maintenance of such behaviors in safe settings, however, may prevent youth from engaging in the formerly described social behaviors necessary for developmental success. Reports from educators of these children in the present resettlement region confirm this finding: their top complaint is children’s school avoidance due to fear of being separated from parents and siblings. Separation anxiety, subsequent disengagement from the educational system, and potential effects of chronic stress and prolonged trauma exposure may be detrimental to cognitive functioning of developing youth. Therapeutic focus on separation anxiety should be a top priority for both care providers and intervention researchers. Creative arts and movement therapies have been found to be successful in addressing separation anxiety in refugee youth and may be considered for use in therapeutic, educational, and community settings (Grasser et al., 2019; Feen-Calligan et al., 2020).

Previous literature indicates that in both children and adults, females are more likely to show more severe anxiety-related symptoms and be diagnosed as either a current or recovered case (Carter et al., 2011). However, our findings in both Iraqi and Syrian refugee youth were not consistent with this; there were no sex differences in self-reported posttraumatic stress or anxiety symptoms.

Finally, we compared our cohort of Iraqi refugee youth to Syrian refugee youth, to investigate whether trauma and stress-related psychopathology differed between these two cohorts, in an effort to showcase the variability in both refugees’ experiences as well as outcomes despite being exposed to potentially similar contexts of civilian war trauma and forced migration. It is critical to note that while the comparison between the Iraqi and Syrian cohorts was a primary aim of this work, the limited influx of Iraqi refugees to the region and the nature of this being an observation study of a convenience sample resulted in an unbalanced sample of youth from the two countries of origin. While such comparative research is highly significant (Kéri, 2015) and should be expanded upon in future research, this is also quite complicated due to not only potentially unbalanced designs and potentially small samples (Kéri, 2015) but also unmeasured between-group differences, such as quality and intensity of traumatic events, sociodemographic factors, and personality.

Unlike our Syrian cohort, Iraqi youth had lower severity of total posttraumatic stress symptoms, re-experiencing symptoms, and negative cognition and mood symptoms, as well as better subjective health. This may be indicative of a higher level of direct trauma and war exposure in Syrian youth. While Iraq has slowly been recovering its economy and infrastructure over the past decade, Syria’s economy and infrastructure continues to deteriorate (United States, Central Intelligence Agency). While in Iraq two major militia groups currently exist, with some associated political parties, in Syria, there are many political and armed opposition groups leading to greater instability and uncertainty in the region (United States, Central Intelligence Agency). Additionally, while youth from both countries had experienced warzone exposure and forced migration, we did not assess other potential adverse events youth may have been exposed to, which may have varied between samples resulting in the observed differences in posttraumatic stress symptoms. The presence of other adversities should be taken into account in both the clinical and research setting when working with refugee youth, as the effects of warzone exposure and forced migration on trauma-related psychopathology may be dependent on exposure to other adversities (Karam et al., 2019).

A prior study of adult migrants, refugees, and asylum seekers from Syria, Afghanistan, and Iraq did not identify significant differences in rates of PTSD across these three groups of individuals (Kéri, 2015); similarly, in our present study of youth, there were some similarities between the two groups – including equitable levels of anxiety-related symptoms based on the SCARED questionnaire and, more notably, equally high level of separation anxiety. This is specifically important as, for migrant children, it is vital to be able to explore the environment, and go to school, to learn the new environment, and its way of living. The possible indication of PTSD was lower than other studies from the Middle East for our cohort of Iraqi refugee youth, and this finding is similar to our finding in Syrian refugee youth (Javanbakht et al., 2018). Both estimates of diagnoses may be lower than expected due to a multitude of potential explanations: (a) assessments were conducted in a safe context – i.e., outside of the country of origin where repeated traumatic events were routinely occurring, and trauma cues were present even in safer environments like refugee camps (Acarturk et al., 2018); (b) assessments were conducted within the first month of resettlement, which, while stressful, could be a more positive time as the long journey to safety is over and there is hope for a new life in a new country; (c) parents could have a role in buffering the impact of trauma in children, and reducing the level to subclinical high anxiety, rather than clinical PTSD (van Rooij et al., 2017); (d) children may have a different developmental course than that of adults and may show a more delayed onset; (e) refugee youth may be resilient and may have formed adaptive coping skills to deal with the stressful experiences of violence and migration (Sleijpen et al.,2016a,b); (f) possible indication of PTSD diagnosis was made based on self-report data, and not full clinical interview, which may lead to inaccuracies in diagnostic predictions. Self-reported posttraumatic stress symptom severity, however, was significantly greater in Syrian refugee youth compared to Iraqi youth.

Comparing the Iraqi and Syrian groups is of particular importance, as our results demonstrate that findings from one refugee population cannot easily be generalized to another, even when individuals are originating from similar regions (i.e., the Middle East, in this case), and even when they resettle in the same geographic location and social environment. Knowing these differences will aid resettlement agencies in determining resources needed for best resettlement practices by country of origin, and may also direct treatment, as intervention outcomes may differ as a factor of ethnicity – which may be a proxy measure for a number of other variant factors. Despite the differences in samples our study has identified, these between-group differences may be explained by other uncontrolled variables in the quality and intensity of traumatic events, sociodemographic factors, and personality, which may or may not be linked to nationality. Our findings suggest that it is critical that mental health screening be included in the initial physical health screening protocol for newly arriving refugees, as early intervention may lead to more positive outcomes (Abu Suhaiban et al., 2019). Since these conditions are among the most highly prevalent health concerns in refugees (Abu Suhaiban et al., 2019), regular screening for them seems reasonable, and potentially efficacious as well as feasible community-based interventions to reduce trauma-related psychopathology in youth may include creative arts and movement therapies, which can be culturally adapted (Grasser et al., 2019; Feen-Calligan et al., 2020). Understanding the regions, contexts, social norms, cultures, and values from which individuals have sought refugee status is also critical, seeing as outcomes differ by ethnicity. If the mental health needs of children, particularly children in conflict, are not attended to and supported, there will be great global health and economic cost (The Lancet, 2019).

### Limitations

This study is limited by a small sample size of refugee youth. The limitations in sampling are critical to note, both in terms of the unbalanced design when comparing the two cohorts – Syrian and Iraqi refugee youth – and when interpreting the findings regarding severity of trauma-related psychopathology (PTSD and anxiety symptoms) in Iraqi refugee youth. These findings cannot be generalized to the refugee population as a whole. Our sample was smaller than anticipated because of reductions to the number of refugees entering the United States. While a larger target sample had been intended, travel bans that went into effect during the recruitment period stopped the flow of new arrivals into the United States. Therefore, due to the limited sample, this cannot be considered a prevalence study; rather, the goal of this study was to assess mental health symptoms in incoming Iraqi refugee youth and compare two refugee groups resettled in Southeastern Michigan at the height of the diaspora from the regions of Syria and Iraq. Here, we signal the importance of different refugee groups – how even those groups that seem very similar coming from neighboring countries facing similar adversities may differ in severity of trauma-related psychopathology at resettlement. The recent lift on travel bans may allow for future, better powered studies to assess the impact of civilian warzone trauma exposure and forced migration on refugee youth, which will be necessary to better power comparative analyses. Despite the challenges to enrollment of the Iraqi refugees resettling in Southeastern Michigan who presented at primary care clinics within the enrollment period, we were able to enroll 88% of the eligible population.

Another limitation is the reliance on self-report questionnaires. Formal diagnoses were not made by a clinician due to time restrictions for the length of the research visit. Self-report questionnaires may be subject to issues of over- and underreporting of symptoms; most research has found that self-report questionnaires overreport symptoms (Charlson et al., 2019). It should be noted that possible indications for PTSD based on the UCLA PTSD RI may not accurately capture possible presence of a disorder given that youth rarely screen positive on this measure because the criteria have been derived from adult populations (Copeland et al., 2007; Cohen et al., 2008; Miller et al., 2019).

### Future Directions

The present cross-sectional study of Iraqi refugee youth indicates greater need for clinical and research focus on refugee health. Like Syrian refugee youth, Iraqi youth have high rates of separation anxiety. Iraqi refugee youth, however, have lower severity of posttraumatic stress symptoms and better subjective health than Syrian refugee youth. These findings show that results from one refugee population are not necessarily generalizable to another, even when they are from the assumed similar region, or are resettled at the same time, and in the same new environment. This study indicates the importance of mental health screening for newly arriving refugee families in order to provide necessary resources, and feasibility of doing so, as it was conducted in a primary care setting where new arrivals were being received. Such screening may reduce the burden of trauma on resettling families, who face ongoing chronic stress, especially given detrimental long-term effects of childhood trauma on physical and mental health during childhood and as adults.

## Data Availability Statement

The datasets presented in this article are not readily available because given the longitudinal nature of this ongoing study, the dataset is not currently available to the public. Requests to access the datasets should be directed to LG, lgrasser@med.wayne.edu.

## Ethics Statement

The studies involving human participants were reviewed and approved by the Institutional Review Board, Wayne State University. Written informed consent to participate in this study was provided by the participants’ legal guardian/next of kin.

## Author Contributions

LG was responsible for the data analysis, interpretation of the results, and preparation of the manuscript. LH led the recruitment efforts. SM served as the protocol manager for the study, and involved in the data collection. SA was involved in the interpretation of the results and assisted with the preparation of the manuscript. CA was in charge of data management and oversaw analysis and also assisted with interpretation of the results and preparation of the manuscript. AJ served as the principal investigator for the study, designed the study, and oversaw interpretation of the results and preparation of the manuscript. All authors contributed to the article and approved the submitted version.

## Conflict of Interest

The authors declare that the research was conducted in the absence of any commercial or financial relationships that could be construed as a potential conflict of interest.
